# Comparison between beta‐blockers and calcium channel blockers in patients with atrial fibrillation according to renal function

**DOI:** 10.1002/clc.24257

**Published:** 2024-04-25

**Authors:** José Antonio Parada Barcia, Sergio Raposeiras Roubin, David González Fernández, André González García, Carla Iglesias Otero, Inmaculada González Bermúdez, Andrés Íñiguez Romo, Emad Abu‐Assi

**Affiliations:** ^1^ Cardiology Department University Hospital Alvaro Cunqueiro Vigo Spain; ^2^ University of Santiago de Compostela Santiago Spain; ^3^ Health Research Institute Galicia Sur Vigo Spain; ^4^ Centro Nacional de Investigaciones Cardiovasculares Madrid Spain

**Keywords:** atrial fibrillation, beta‐blocker, chronic kidney disease, nondihydropyridine calcium channel blockers

## Abstract

**Background:**

Rate control is the most commonly employed first‐line management strategy for atrial fibrillation (AF) in patients with chronic kidney disease (CKD). Principal agents used to control heart rate (HR) include beta‐blockers (BB) and nondihydropyridine calcium channel blockers (ND‐CCB). However, there is a paucity of published studies of the differences between those drugs in CKD patients.

**Hypothesis:**

The present study aimed to investigate the differences, in terms of hospitalizations due to a poor HR control, in patients with AF under a rate‐control strategy according to glomerular filtration rate (GFR).

**Methods:**

The study cohort included 2804 AF patients under rate‐control regime (BB or ND‐CCB) between January 2014 and April 2020. The end point, determined by competing risk regression, was hospitalizations for AF with rapid ventricular response (RVR), slow ventricular response (SVR), and need for pacemaker.

**Results:**

On multivariate analysis, there were no statistical differences between ND‐CCB and BB for subjects with GFR > 60 mL/min/1.73 m^2^ (subdistribution heart rate [sHR] 0.850, 95% confidence interval [CI]: 0.61–1.19; *p* = .442) and GFR 30–59 mL/min/1.73 m^2^ (sHR 1.242, 95% CI: 0.80–1.63; *p* = .333), while in patients with GFR < 30 mL/min/1.73 m^2^, ND‐CCB therapy was associated with increased hospitalizations due to poor HR control (sHR 4.53, 95% CI: 1.19–17.18; *p* = .026).

**Conclusion:**

In patients with GFR ≥ 30 mL/min/1.73 m^2^, the choice of ND‐CCB or BB had no impact on hospitalizations due to poor HR control, while in GFR < 30 mL/min/1.73 m^2^, a possible association was detected. The effects of these drugs on GFR < 30 mL/min/1.73 m^2^ would require further investigation.

## Key points

- Rate control is the most commonly employed first‐line management strategy for atrial fibrillation in patients with chronic kidney disease. Evidence to guide selection between beta‐blockers and nondihydropyridine calcium channel blockers is scarce.
- This is the first study to compare patients with atrial fibrillation under rate‐control strategy according to renal function.
- In patients with glomerular filtration rate (GFR) ≥ 30 mL/min/1.73 m^2^, both nondihydropyridine calcium channel blockers (ND‐CCB) and beta‐blockers therapy have good results in heart rate (HR) control, while in advanced chronic kidney disease (GFR < 30 mL/min/1.73 m^2^), a possible association of ND‐CCB with increased hospitalizations due to poor HR control was detected.

## 1 INTRODUCTION

Atrial fibrillation (AF) and chronic kidney disease (CKD) frequently coexist, as both share risk factors and pathophysiologic mechanisms.^1^ Patients with CKD have a threefold increased risk of AF compared with the healthy population.^2^ In fact, recent registry data suggest that there is a close bidirectional relationship between both conditions.^3^ Heart rate (HR) control is the most commonly employed first‐line management strategy for AF in patients with CKD.^4^ Excessive ventricular rates during AF might cause severe symptoms, such as palpitations, dyspnea and fatigue. Even, in some patients, it can lead to the development of a tachycardia‐induced cardiomyopathy and may worsen congestive heart failure (HF), increasing the risk of hospital admission and mortality.^5^

Strategies targeted at reducing the ventricular rate during AF rely on agents that work by prolonging atrioventricular node refractoriness. Principal classes of agents used include beta‐adrenergic blockers (BB) and nondihydropyridine calcium channel blockers (ND‐CCB). There is little evidence to support selection of HR control therapy in patients with AF, in particular those with coexisting CKD.

Despite the high prevalence of AF in patients with CKD, there is a paucity of published studies of the clinical value and differences between those drugs in this context. Furthermore, CKD patients are commonly underprescribed recommended cardiovascular medications.^6^

The present study aimed to investigate the differences, in terms of hospitalizations due to a poor HR control, in patients with AF under a rate‐control strategy according to glomerular filtration rate (GFR).

## 2 METHODS

### 2.1 Study population

**Figure 1:**
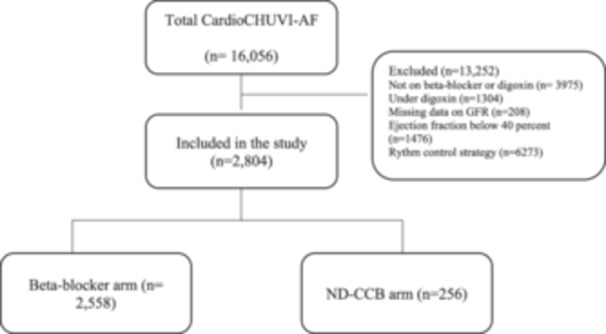
Study flow diagram. GFR, glomerular filtration rate; ND‐CCB, nondihydropyridine calcium channel blockers.

This is a retrospective observational study conducted in a subset of participants treated with BB or ND‐CCB of the CardioCHUVI‐AF registry (Registry of Atrial Fibrillation from University Hospital of Vigo, NCT‐04364516). AF patients were identified through administrative databases, using the Galician Healthcare Service information system. Electronic medical records were analyzed to collect data on baseline clinical variables, treatment, and follow‐up events. Pharmacotherapy was determined by means of filled prescriptions. The CardioCHUVI‐AF registry included 16,056 patients from the health area of Vigo (Galicia, Spain) with a confirmed diagnosis based on an electrocardiogram between January 2014 and April 2020. Patients without data regarding to GFR (*n* = 208), those who received digoxin (*n* = 1304) and those with left ventricular ejection fraction (LVEF) below 40% (*n* = 1476) were excluded from this analysis; we also excluded 6273 patients under a rhythm‐control strategy and 3975 subjects who were not taking any treatment (nor rhythm‐control nor rate‐control). Thereby, the final study group consisted of 2804 patients (Figure 1). The entire data handling process complied with ethical and legal standards, in particular with Declaration of Helsinki and was approved by the local ethics committee. All the data were collected and processed and were anonymized by a code. Informed consent was not required for the present study.

### 2.2 Follow‐up, definition, and study outcomes

Follow‐up was performed from the first time the patient was medically assessed for AF between January 2014 and April 2020 and lasted until September 2020. Patients remained in the analysis until an event occurred or until the end of follow‐up.

Renal status was determined at baseline. Estimated GFR was calculated according to Chronic Kidney Disease Epidemiology Collaboration (CKD‐EPI) equation, which estimates GFR from age, sex, and serum creatinine.^7^ The closest creatinine value to the date of inclusion was gathered. The sample was classified according to KDIGO GFR (G) categories, after combining them into three groups: Nonrenal disease, which included CKD G1 (eGFR ≥ 90 mL/min/1.73 m^2^) and G2 (60–89 mL/min/1.73 m^2^); nonadvanced CKD, which incorporated G3a (eGFR 45–59 mL/min/1.73 m^2^), G3b (30–44 mL/min/1.73 m^2^); and advanced CKD, which involved G4 (15–29 mL/min/1.73 m^2^) and G5(<15 mL/min/1.73 m^2^ or on hemodialysis).^8^

A rapid ventricular response (RVR) episode was defined as a HR over 120 beats per minute.^9^ A slow ventricular response (SVR) episode was defined as an HR below 40 beats per minute.

The end point of the study was admissions due to poor heart rate control, defined as hospitalizations for AF with RVR, hospitalizations for AF with SVR, and need for pacemaker.

### 2.3 Statistical analysis

Baseline characteristics were described using frequencies and percentages for categorical data, and mean ± standard deviation for continuous data. Differences in characteristics were determined using *χ* ^2^ tests and unpaired *t* tests, respectively. Normality was analyzed with Shapiro–Wilk test. The risk of admissions due to a poor HR control was determined by competing risk regression using the fine and gray model, considering death as a competing risk. Fine and gray models were fitted by GFR. Multivariate adjustment was developed including all those variables with clinical significance and those that had been associated with higher risk of the combined end point of in the univariate analysis (Supporting Information S1: Table 1). Results were reported as subdistribution HR (sHR) and 95% confidence interval (CI) and were graphically represented with cumulative incidence curves. All *p* values < .05 were accepted as statistically significant. All statistical analyses were performed using Stata 16.1.

## 3 RESULTS

A total of 2804 patients (82.0 ± 4.8 years, 62.2% women) were followed up. The baseline characteristics are presented in Table 1.

**Table 1:** Baseline characteristics of the patients.

Characteristics	Beta‐blocker (*n* = 2558)	ND‐CCB (*n* = 246)	*p* Value
Age, years	82.15 (4.74)	80.16 (5.08)	.000
Female sex, *n* (%)	1604 (62.71)	139 (56.50)	.055
GFR, mL/min/1.73 m^2^	63.35 (18.65)	68.13 (17.63)	.000
Admission for congestive heart failure, *n* (%)	211 (8.25)	16 (6.50)	.338
Permanent AF, *n* (%)	1880 (73.49)	190 (77.24)	.202
COPD, *n* (%)	244 (9.54)	49 (19.92)	.000
Hypertension, *n* (%)	2066 (80.77)	185 (75.20)	.036
Diabetes mellitus, *n* (%)	539 (21.07)	43 (17.48)	.185
Dyslipidemia, *n* (%)	1293 (50.55)	145 (58.94)	.012
BMI	30.03 (4.36)	30.34 (4.45)	.289
Peripheral artery disease, *n* (%)	98 (3.83)	9 (3.66)	.893
Ischemic heart disease, *n* (%)	393 (15.36)	36 (14.63)	.761
CHA2DS2Vasc scale	4.08 (1.10)	3.96 (1.10)	.080
HAS‐BLED	2.94 (1.09)	2.90 (1.16)	.596
Anemia, *n* (%)^a^	623 (24.35)	43 (17.48)	.016
Dementia, *n* (%)	251 (9.81)	32 (13.01)	.112
Aortic stenosis, *n* (%)	129 (5.04)	11 (4.47)	.694
Mitral regurgitation, *n* (%)	86 (3.36)	10 (4.07)	.562
Anticoagulated, *n* (%)	2206 (86.24)	211 (85.77)	.839
Statin, *n* (%)	1173 (45.86)	136 (55.28)	.005
ACE or ARB, *n* (%)	1462 (57.15)	132 (53.66)	.290

*Note*: Values are mean ± standard deviation or *n* (%).

Abbreviations: ACE, angiotensin‐converting enzyme inhibitor; ARB, angiotensin receptor blocker; BMI, body mass index; CHA2DS2Vasc, congestive heart failure, hypertension, age ≥ 75 years, diabetes mellitus, previous stroke, transient ischemic attack, or thromboembolism, vascular disease, age 65–74 years, sex; COPD, chronic obstructive pulmonary disease; GFR, glomerular filtration rate; HAS‐BLED, hypertension, abnormal renal function, abnormal liver function, stroke, bleeding, labile international normalized ratio, elderly, drug therapy, alcohol intake, nonsteroidal anti‐inflammatory drug; ND‐CCB, nondihydropyridine calcium channel blockers.

^a^ Anemia at inclusion.

BB were the most used drugs (BB 91.2% vs. ND‐CCB 8.8%). Patients under BB tend to be older (82 vs. 80 years, *p* = .000) and had more hypertension than those treated with ND‐CCB. The use of anticoagulation therapy was comparable between both groups (BB 86.2% vs. ND‐CCB 85.8%).

As GFR decreased, patients were more frequently treated with BB. In addition, 1197 patients (88.5%) with GFR > 60 mL/min/1.73 m^2^, 1198 (93.6%) with GFR 30–59 mL/min/1.73 m^2^ and 163 (95.1%) with GFR < 30 mL/min/1.73 m^2^ were under treatment with BB, respectively (more information in Supporting Information S1: Table 2).

During a mean follow‐up of 3.27 ± 1.88 years, 757 patients died (27.0%) and 723 (25.8%) had an admission due to poor HR control: 217 were hospitalized for AF with SVR (7.7%), 434 for AF with RVR (15.5%), and 72 patients needed urgent pacemaker (2.6%).

**Figure 2:**
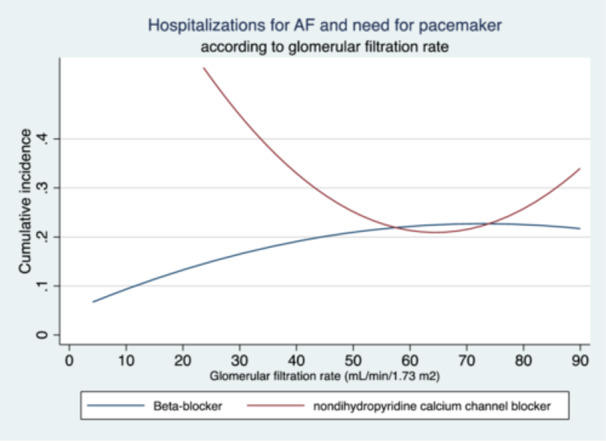
Incidence of hospitalizations due to poor heart rate control in patients with AF according to glomerular filtration rate. AF, atrial fibrillation.

The incidence of hospitalizations increased among patients who received ND‐CCB as baseline GFR declined (Figure 2).

Table 2 shows the results from fine and gray multivariate adjustment of model of the combined end‐point risk. On multivariate analysis, there were no statistical differences between ND‐CCB and beta‐blocker therapy for subjects with GFR > 60 mL/min/1.73 m^2^ (sHR 0.850, 95% CI 0.61–1.19; *p* = .442) and GFR 30–59 mL/min/1.73 m^2^ (sHR 1.242, 95% CI 0.80–1.63; *p* = .333), while in patients with GFR < 30 mL/min/1.73 m^2^, ND‐CCB were associated with increased hospitalizations due to poor HR control (sHR 4.53, 95% CI 1.19–17.18; *p* = .026).

**Table 2:** Subhazard ratios for the risk of hospitalizations due to poor heart rhythm control (admissions for AF with RVR, AF with SVR, and need for pacemaker).

	GFR ≥ 60 mL/min/1.73 m^2^ (*N* = 1353)		GFR 30–59 mL/min/1.73 m^2^ (*N* = 1280)		GFR < 30 mL/min/1.73 m^2^ (*N* = 163)	
Characteristics	sHR	*p* Value	sHR	*p* Value	sHR	*p* Value
ND‐CCB	0.85 (0.61–1.19)	.342	1.24 (0.80–1.93)	.333	4.53 (1.19–17.18)	.026
Age, years	0.93 (0.89–0.97)	.000	0.94 (0.91–0.98)	.001	0.95 (0.87–1.03)	.243
Female sex	1.38 (1.49–1.75)	.008	1.31 (0.98–1.76)	.069	1.04 (0.26–4.19)	.957
Permanent AF	0.77 (0.60–0.99)	.039	0.583 (0.44–0.77)	.000	0.40 (0.13–1.25)	.115
BMI	0.99 (0.96–1.01)	.307	0.99 (0.96–1.03)	.626	0.89 (0.79–1.02)	.082
Alcohol abuse	0.94 (0.70–1.26)	.697	1.55 (1.10–2.19)	.013	3.12 (0.29–33.10)	.345
Diabetes mellitus	1.06 (0.82–1.39)	.627	1.12 (0.82–1.52)	.494	1.16 (0.37–3.60)	.797
Dyslipidemia	0.82 (0.42–1.59)	.556	0.93 (0.54–1.59)	.792	3.06 (0.56–16.71)	.196
COPD	1.52 (1.40–2.13)	.015	1.27 (0.86–1.88)	.222	0.83 (0.08–9.01)	.876
Left atrial dilatation	1.05 (0.96–1.16)	.270	1.10 (0.97–1.24)	.129	1.15 (0.72–1.82)	.563
Prior admission for congestive heart failure	2.62 (2.48–3.30)	.000	2.54 (1.94–3.34)	.000	2.62 (2.09–3.30)	.000
Dementia	1.26 (0.92–1.73)	.144	1.30 (0.90–1.90)	.166	1.26 (0.92–1.73)	.144
ACE or ARB	1.07 (0.85–1.35)	.539	1.11 (0.85–1.44)	.441	1.07 (0.85–1.35)	.539
Aortic stenosis	1.25 (0.84–1.88)	.271	0.91 (0.56–1.50)	.717	0.43 (0.12–1.53)	.190
Mitral regurgitation	1.67 (1.00–2.80)	.051	1.07 (0.61–1.87)	.822	8.13 (1.53–43.34)	.014
Statins	1.28 (0.66–2.48)	.465	1.07 (0.62–1.84)	.813	0.83 (0.15–4.61)	.829

*Note*: Adjusted by age, rate‐control strategy, female sex, diabetes, ACE or ARB, aortic stenosis, BMI, alcohol abuse, dyslipidemia, COPD, mitral regurgitation, statin, dilatation of the left atrium, previous admission for congestive heart failure, dementia, and permanent AF.

Abbreviations: ACE, angiotensin‐converting enzyme inhibitor; AF, atrial fibrillation; ARB, angiotensin receptor blocker; BMI, body mass index; COPD, chronic obstructive pulmonary disease; CHA2DS 2‐VASc, congestive heart failure; hypertension, age ≥75 years, diabetes mellitus, previous stroke, transient ischemic attack, or thromboembolism, vascular disease, age 65–74 years, sex; GFR, glomerular filtration rate; HAS‐BLED, hypertension, abnormal renal function, abnormal liver function, stroke, bleeding, labile INR, labile international normalized ratio, elderly, drug therapy, alcohol intake, and nonsteroidal anti‐inflammatory drug; ND‐CCB, nondihydropyridine calcium channel blockers; RVR, rapid ventricular response; sHR, subdistribution heart rate; SVR, slow ventricular response.

## 4 DISCUSSION

In this registry study of prospectively recruited patients with AF, we observed that in patients with GFR ≥ 30 mL/min/1.73 m^2^, both ND‐CCB and BB therapy have good results in HR control. Second, in patients with advanced CKD (GFR <30 mL/min/1.73 m^2^) the use of ND‐CCB over BB was associated with increased admissions due to poor HR control. These findings are likely to be relevant, as, despite the fact that BB and ND‐CCB have been used as the cornerstone of HR control therapy for AF patients for decades, evidence to guide selection between both drugs is scarce. Most of the previous studies that compared the effectiveness of different rate‐controlling therapies were small, and often compared drugs with multiple dose regimens combined with digitalis.^10^

To the best of our knowledge, this is the first study to compare AF patients under rate‐control strategy according to renal function. CKD patients, through several mechanisms including myocardial fibrosis and augmented inflammation, have a greater risk of suffering from AF and HR control is the most commonly employed first‐line management strategy in CKD patients, although there were no previous studies comparing outcomes in this population.

The evidence on rate control in AF management is limited. ESC guidelines include a Class II recommendation for lenient rate control (<110 bpm) in asymptomatic patients with AF,^11^ based on the results of the RACE II trial, which showed that a lenient rate‐control treatment strategy (<110 bpm) was noninferior to a strict rate‐control treatment strategy (<80 bpm) in terms of symptoms control and mortality.^12^ No drug‐specific subanalysis had been performed. To evaluate the efficacy between BB and ND‐CCB, Ulimoen et al. promoted the Rate Control in Atrial Fibrilla (RATAF) study, which compared four rate‐controlling drugs (BB and ND‐CCB) in only 60 symptomatic patients with permanent AF without heart failure.^13^ As well as in the RACE II trial, patients included in the RATAF study were relatively young (71 ± 9 years), had low comorbidity burden and no data regarding to GFR have been shown. Both diltiazem, which seemed to be the most effective in reducing the ventricular rate, and verapamil, preserved exercise capacity, reduced arrhythmia‐related symptoms, and reduced levels of NT‐pro‐B‐natriuretic peptide, but not BB. Those positive effects of ND‐CCB and the fact that recent studies have suggested that BB use in both HFpEF and AF may increase the risk for HF,^14^ had led Meyer et al. to conduct a large retrospective cohort analysis, comparing ND‐CCB and BB in patients with AF and heart failure, with controversial results.^15^ Compared with BB, ND‐CCB were associated with fewer HF admissions but also with more all‐cause deaths. No subgroup analysis according to GFR has been made in this study. Randomized studies that compare the two medication classes have not been performed yet. While the pharmacokinetics of ND‐CCB is unaltered in patients with CKD,^16^ BB are renally or hepatically cleared depending on the type.^17^ Even, ND‐CCB have been shown to possess an antiproteinuric effect that could be particularly relevant in CKD. In our study, in patients with GFR ≥ 30 mL/min/1.73 m^2^, the use of ND‐CCB over BB therapy was not associated with increased hospitalizations due to poor HR control.

Although equally recommended in the ESC guidelines, ND‐CCB are less often used than BBs. In fact, ND‐CCB are not very popular in Europe. This wide consensus is particularly surprising considering previous data. In the Italian survey on drug management of AF conducted by Diemberger et al., only 14% of clinicians considered ND‐CCB as the first choice in CKD, a result which is higher than the registered in our cohort.^18^

Rate control may result in slow ventricular rates or heart block. In the RACE II trial, the need for pacemaker was 1.4% at 3 years in the strict rate‐control arm (similar to the 0.8% observed in the lenient rate‐control arm), despite the possibility to combine BB plus ND‐CCB (used in more than one‐fifth of the patients). It has been reported that in patients with CKD, major ECG abnormalities are frequently present.^19^ Our cohort included older patients than the RACE II trial, with a bigger burden of comorbidities and almost 52% of them had CKD, which could explain the higher implantation rate of pacemaker (2.57%). Even so, the implantation rate of pacemakers in this cohort was low.

The possible association of ND‐CCB with increased hospitalization due to poor HR control in patients with advanced CKD is underpowered and it should be endorsed in other studies.

### 4.1 Limitations

There are some limitations inherent to the retrospective study design and the subsequent bias. The two patient groups (BB and ND‐CCB) were of unequal size. Outcomes were analyzed according to treatment at baseline and treatment changes were not accounted for in this study. Furthermore, there was a lack of details (e.g., dose and type) on the prescription of ND‐CCB and BB (despite the fact that the most common BB in our area is bisoprolol, we do not have data in this cohort).

## 5 CONCLUSIONS

In patients with GFR ≥ 30 mL/min/1.73 m^2^, the choice of ND‐CCB or BB had no impact of hospitalizations due to poor HR control, while in advanced CKD (GFR < 30 mL/min/1.73 m^2^), a possible association of ND‐CCB with increased hospitalizations due to poor HR control was detected. The effects of these drugs on GFR < 30 mL/min/1.73 m^2^ would require further investigation.

## Supporting information

Supporting information.

## Data Availability

The data that support the findings of this study are available from the corresponding author upon reasonable request.
