# Supplementary material for: Comparison between beta‐blockers and calcium channel blockers in patients with atrial fibrillation according to renal function
Source: Clin Cardiol. 2024 Apr 25;47(5):e24257. doi: 10.1002/clc.24257 (PMC11046037; doi:10.1002/clc.24257)
Supplement: Supplementary file 1 — Supporting information. [file CLC-47-e24257-s001.docx]

| Supplemental table 1. Univariate analysis for the risk of hospitalizations due to poor heart rhytm control (admissions for AF with RVR, AF with SVR and need for pacemaker) | | |
| --- | --- | --- |
| Characteristics | Univariate analysis | |
|  | sHR | P-value |
| ND-CCB | 1.122 (0.875-1.439) | 0.363 |
| GFR 30-60 | 0.778 (0.667-0.907) | 0.001 |
| GFR <30 | 0.621(0.425-0.908) | 0.014 |
| Age — yrs | 0.929 (0.912-0.945) | 0.000 |
| Female sex — no. (%) | 1.166(1.000-1.361) | 0.051 |
| Permanent AF— no. (%) | 0.725 (0.615-0.855) | 0.000 |
| Alcohol abuse— no. (%) | 1.392 (1.145-1.693) | 0.001 |
| Diabetes mellitus— no. (%) | 1.278 (1.071-1.524) | 0.006 |
| Hypertension— no. (%) | 0.960 (0.803-1.148) | 0.658 |
| Smoking habit— no. (%) | 0.957 (0.548-1.674) | 0.879 |
| Hepatopathy— no. (%) | 0.933 (0.574-1.512) | 0.779 |
| Dislipemia— no. (%) | 1.251 (1.076-1.454) | 0.004 |
| Previous cancer — no. (%) | 1.053 (0.791-1.401) | 0.725 |
| Aortic stenosis— no. (%) | 1.395 (1.046-1.861) | 0.023 |
| Mitral regurgitation — no. (%) | 1.895 (1.389-2.585) | 0.000 |
| Admission for heart failure— no. (%) | 0.978 (0.730-1.308) | 0.879 |
| Congestive heart failure — no. (%) | 2.514 (2.164-2.921) | 0.000 |
| Ischemic heart disease— no. (%) | 0.986 (0.801-1.213) | 0.896 |
| COPD — no. (%) | 1.397 (1.116-1.748) | 0.004 |
| CONUT score— no. (%) | 1.041 (0.993-1.090) | 0.096 |
| EF <40% — no. (%) | 1.557 (1.218-1.990) | 0.000 |
| Atrial dilatation — no. (%) | 1.219 (1.149-1.294) | 0.000 |
| BMI— no. (%) | 1.012 (0.998-1.034) | 0.091 |
| Dementia— no. (%) | 1.331 (1.069-1.656) | 0.010 |
| ACE or ARB— no. (%) | 1.201 (1.029-1.401) | 0.020 |
| Anticoagulated— no. (%) | 1.199 (0.952-1.511) | 0.124 |
| Statins — no. (%) | 1.244 (1.071-1.444) | 0.004 |
| Values are mean +/- SD or n (%).  Abbreviations: **AF= atrial fibrillation; GFR= glomerular filtration rate; BMI= body mass index; COPD= chronic obstructive pulmonary disease; EF= ejection fraction; ACE= angiotensin-converting enzyme inhibitor; ARB= angiotensin receptor blocker; ND-CCB= nondihydropyridine calcium channel blockers** | | |

| Supplemental table 2. Baseline characteristics according to GFR | | | | | | | | | |
| --- | --- | --- | --- | --- | --- | --- | --- | --- | --- |
| Characteristics | GFR  >60 mL/min/1.73 m^2^ | | | GFR  30-59 mL/min/1.73 m^2^ | | | GFR  <30 mL/min/1.73 m^2^ | | |
|  | Beta-blocker  (n= 1,197) | ND-CCB  (n= 156) | p Value | Beta-blocker  (n= 1,198) | ND-CCB  (n= 82) | p Value | Beta-blocker  (n= 163) | ND-CCB  (n= 8) | p Value |
| Age - y | 80.12 (3.65) | 78.52 (2.86) | 0.000 | 83.61 (4.73) | 82.25 (6.26) | 0.014 | 86.30 (5.17) | 90.83 (5.43) | 0.017 |
| Female sex- no. (%) | 654 (54.64) | 82 (52.56) | 0.625 | 828 (69.12) | 51 (62.20) | 0.191 | 122 (74.85) | 6 (75.00) | 0.992 |
| Admission for congestive heart failure -no. (%) | 71 (5.93) | 7 (4.49) | 0.467 | 113 (9.43) | 7 (8.54) | 0.788 | 27 (16.56) | 2 (25.00) | 0.535 |
| Hypertension -no. (%) | 962 (80.37) | 118 (75.64) | 0.166 | 970 (80.97) | 60 (73.17) | 0.085 | 134 (82.21) | 7 (87.50) | 0.701 |
| Diabetes mellitus – no. (%) | 253 (21.04) | 25 (16.03) | 0.137 | 247 (20.62) | 18 (21.95) | 0.773 | 39 (23.93) | 0 (0) | 0.115 |
| Ischemic heart disease - no. (%) | 178 (14.87) | 24 (15.38) | 0.865 | 175 (14.61) | 9 (10.98) | 0.364 | 40 (24.54) | 3 (37.50) | 0.409 |
| CHA2DS2Vasc scale | 3.93 (1.06) | 3.81 (0.98) | 0.171 | 4.18 (1.09) | 4.16 (1.17) | 0.861 | 4.50 (1.31) | 4.75 (1.83) | 0.861 |
| HAS-BLED | 2.84 (1.05) | 2.90 (1.18) | 0.560 | 2.97 (1.10) | 2.88 (1.12) | 0.448 | 3.42 (1.22) | 3.25 (1.28) | 0.448 |
| Anemia- no. (%) * | 201 (16.79) | 20 (12.82) | 0.207 | 342 (28.55) | 18 (21.95) | 0.199 | 80 (49.08) | 5 (62.50) | 0.459 |
| Aortic stenosis- no. (%) | 57 (4.76) | 6 (3.85) | 0.610 | 62 (5.18) | 5 (6.10) | 0.717 | 10 (6.13) | 0 (0) | 0.470 |
| Mitral regurgitation- no. (%) | 35 (2.92) | 6 (3.85) | 0.527 | 45 (3.76) | 4 (4.88) | 0.609 | 6 (3.68) | 0 (0) | 0.581 |
| Anticoagulated- no. (%) | 1039 (86.80) | 139 (89.10) | 0.420 | 1040 (86.81) | 66 (80.49) | 0.106 | 127 (77.91) | 6 (75.00) | 0.847 |
| Statin- no. (%) | 596 (49.79) | 89 (57.05) | 0.088 | 512 (42.74) | 44 (53.66) | 0.054 | 65 (39.88) | 3 (37.50) | 0.893 |
| ACE or ARB- no. (%) | 730 (60.99) | 86 (55.13) | 0.160 | 658 (54.92) | 41 (50.00) | 0.386 | 74 (45.40) | 5 (62.50) | 0.344 |
| * Anemia at inclusion  Values are mean +/- SD or n (%).  Abbreviations: **ACE= angiotensin-converting enzyme inhibitor; ARB= angiotensin receptor blocker; CHA2DS2Vasc= Congestive Heart failure, Hypertension, Age ≥ 75 years, Diabetes mellitus, previous stroke, transient ischemic attack, or thromboembolism, Vascular disease, Age 65 to 74 years, Sex; GFR= glomerular filtration rate; HAS-BLED= hypertension, abnormal renal function, abnormal liver function, stroke, bleeding, labile INR, elderly, drug therapy, alcohol intake, nonsteroidal anti-inflammatory drug.** | | | | | | | | | |
